# Analysis and comparison of web searches in Google Trends in the business and industrial category for the word’s “resilience” and “wellbeing" during COVID-19

**DOI:** 10.1186/s13104-022-06249-z

**Published:** 2022-12-12

**Authors:** Hang Truong, Craig Steven McLachlan

**Affiliations:** 1 Newcastle Business School, College of Human and Social Futures, University of Newcastle, Callaghan, Australia; 2 Centre for Healthy Futures, Torrens University Australia, 17/51 Foveaux St, Sydney, Surry Hills NSW 2010 Australia

**Keywords:** Wellbeing, Resilience, Search volume index, Google Trends

## Abstract

**Objective:**

The comparison of Google internet searches for worker wellbeing and resilience during COVID has not previously been undertaken. It is important to understand interest in wellbeing and resilience as both constructs influence health and burnout. Our objective to investigate internet interest in both wellbeing and resilience during COVID. Using Google Trends, data on global search English word queries we compared “worker wellbeing” or “wellbeing” versus “resilience” or “psychological resilience”. Two time periods were compared, the last 5 years and the last 12 months, both up until the end of April 2022. The relationship between web search interest, reflected by search volume index (SVI) for all categories versus the business and industrial category evaluated.

**Results:**

Open category searches on Google trends for the key words “worker wellbeing” or “wellbeing” demonstrated increased SVI peaks for COVID periods. Sub-group analyses demonstrated the category business and industrial had less web search interest in wellbeing and an increase in search terms related to resilience but not psychological resilience. Online interest in wellbeing and resilience represents a complex search metric. There are differing search interests depending on whether the category business and industrial is chosen versus the general Google Trends category.

## Introduction

Wellbeing includes components of mental, physical, and work-related health. Changes in work environments, have been recognized as a cause of burnout, stress, and poor worker wellbeing. Decline in wellbeing may be mitigated by improving psychological resilience [1]. However, increasing resilience alone is likely not enough to mitigate a risk of decline in wellbeing, as many internal and external factors can influence individual wellbeing [2].

Resilience can be subdivided into; (i) organizational worker responses to external threats such as COVID; (ii) organizational reliability of employees; (iii) employee adaptability; and (iv) ability of workers to shift business models to maintain services or supply chains [3–5]. Psychological resilience for an individual worker is complex and includes personality traits as well as more trainable intrapersonal factors for coping [5]. It is suggested that information on resilience can be sourced from the internet to inform workplace management of strategies and interventions to enhance workplace resilience.

Declined worker resilience during COVID increases the risk for emotional distress and burnout [1]. Stress affects cognitive performance and worker productivity and self-wellbeing [6]. Resilience allows for the self-mitigation of negative emotions related to workplace stress. Importantly, leadership can offer training to support workplace resilience [7]. Interestingly, it remains unknown whether there has been interest in improving both the wellbeing and resilience of employees during the COVID-19 pandemic.

The internet is a common source of online health information. Public (general collective internet community) versus business and industrial interest in online web search interest in the wellbeing and resilience of workers can be assessed by trends in popular web search engines, such as Google. Analysis of these trend data could allow for real-time surveillance of interest in topics relevant to employee resilience, and wellbeing in countries aware of these English terms. The aims of this paper are to determine the role of searching of internet web browser traffic for worker wellbeing and resilience.

## Main text

### Materials and methods

#### Google trends data availability

Google Trends is an open-source application provided by GOOGLE that provides online infordemiology for selected keyword searchers and or topics over time and geolocation [8]. Google Trends has been increasingly utilized for COVID and mental health research. Search activity data and graph outputs depict the GOOGLE search volume index (SVI), which is normalized over a specific time and or geographic location to improve comparability between search terms. The graphs are scaled on the y axis over a range of 0–100, where 100 is the maximum search interest for the time and location specified. A score of 0 means there were not enough internet search data for this period or periods to reach above an arbitrary zero threshold.

#### Search process and data retrieval

Online interest in resilience and wellbeing was analysed using Google Trends. The keyword “wellbeing” or “worker wellbeing” compared to “resilience” or “psychological resilience” or “worker resilience” was entered in the Google Trends main page (available at: http://www.google.com/trends, accessed 20th May 2022). We set the following filters for the analyzed queries: “Worldwide,” in “All categories” and compared to “business and industrial category” and restricted all internet traffic for “Web searches.” We collected SVIs on a 5 year and 1 year basis dating back from April 30th 2022. The earliest time with available data from Google Trends to April 30, 2021. Note that based on the English words we have chosen, the response from the worldwide searches are likely to be restricted to those countries where these English words are recognized.

#### Data analysis

We qualitatively reported the changes over time of search queries and made reference to geographical changes that were relevant to countries using these English words. We analysed SVI’s over time that corresponded to COVID and non-COVID periods of time reported in Google Trends. We also reviewed wellbeing compared to worker resilience using the predefined terms above, for both relevant countries and categories.

#### Ethical considerations

This study involved aggregated non-identifiable web traffic using summary data on google searches using Google trends. Hence, the design of the study did not require Research Ethics approval.

## Results

### General category Google Trends

**Fig. 1:**
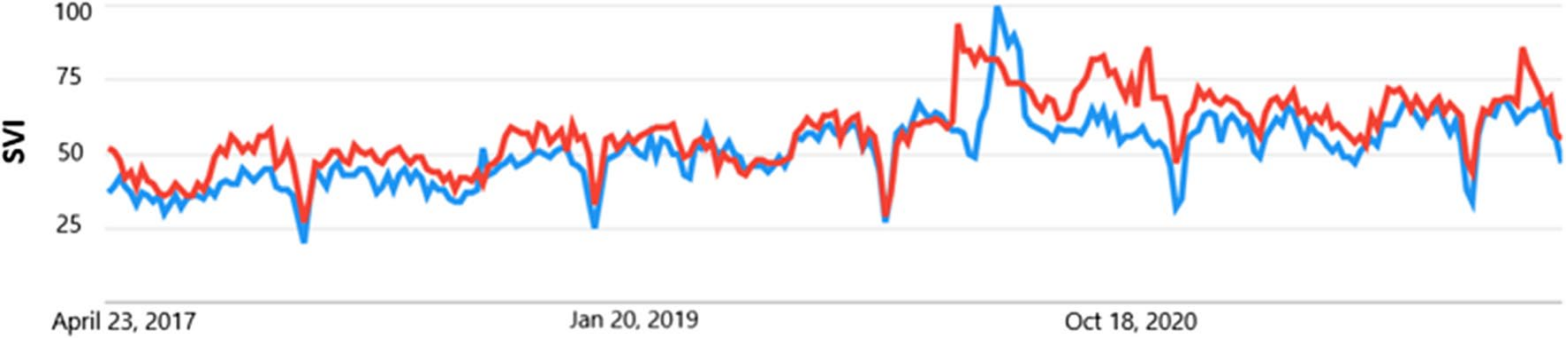
Google trend global web searches for “wellbeing” compared to “resilience” for the last 5 years. The y axis is the SVI on an arbitrary scale set by Google from 0 to 100 and the x axis is the time scale. Blue line is wellbeing and red line is resilience

Interest for general search terms wellbeing compared to resilience for the last 5 years (globally) showed an SVI increase for both search terms in the general category during the COVID period compared to pre-COVID period (see Fig. 1). There was also a slight increase in SVI for resilience during COVID compared to wellbeing (see Fig. 1). There was more interest in wellbeing compared to resilience from four identified countries, including the UK, Ireland, New Zealand and Australia compared to other global nations.

**Fig. 2:**
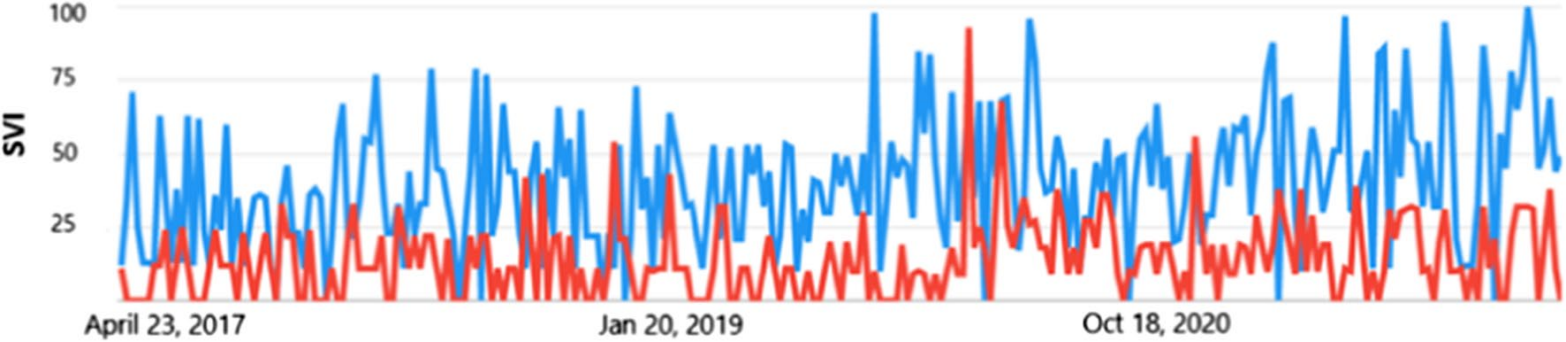
Google trend global web searches for “worker wellbeing” compared to “worker resilience” for the last 5 years. The y axis is the SVI on an arbitrary scale set by Google from 0 to 100 and the x axis is the time scale. Blue line is wellbeing and red line is resilience

When the terms “worker wellbeing” was contrasted to “worker resilience” there was an increase in interest in worker wellbeing, however wellbeing interest appeared to be driven by two global countries the UK and Australia. The increase in SVI was higher during the COVID period (see Fig. 2).

### Business and industrial category for Google Trends

**Fig. 3:**
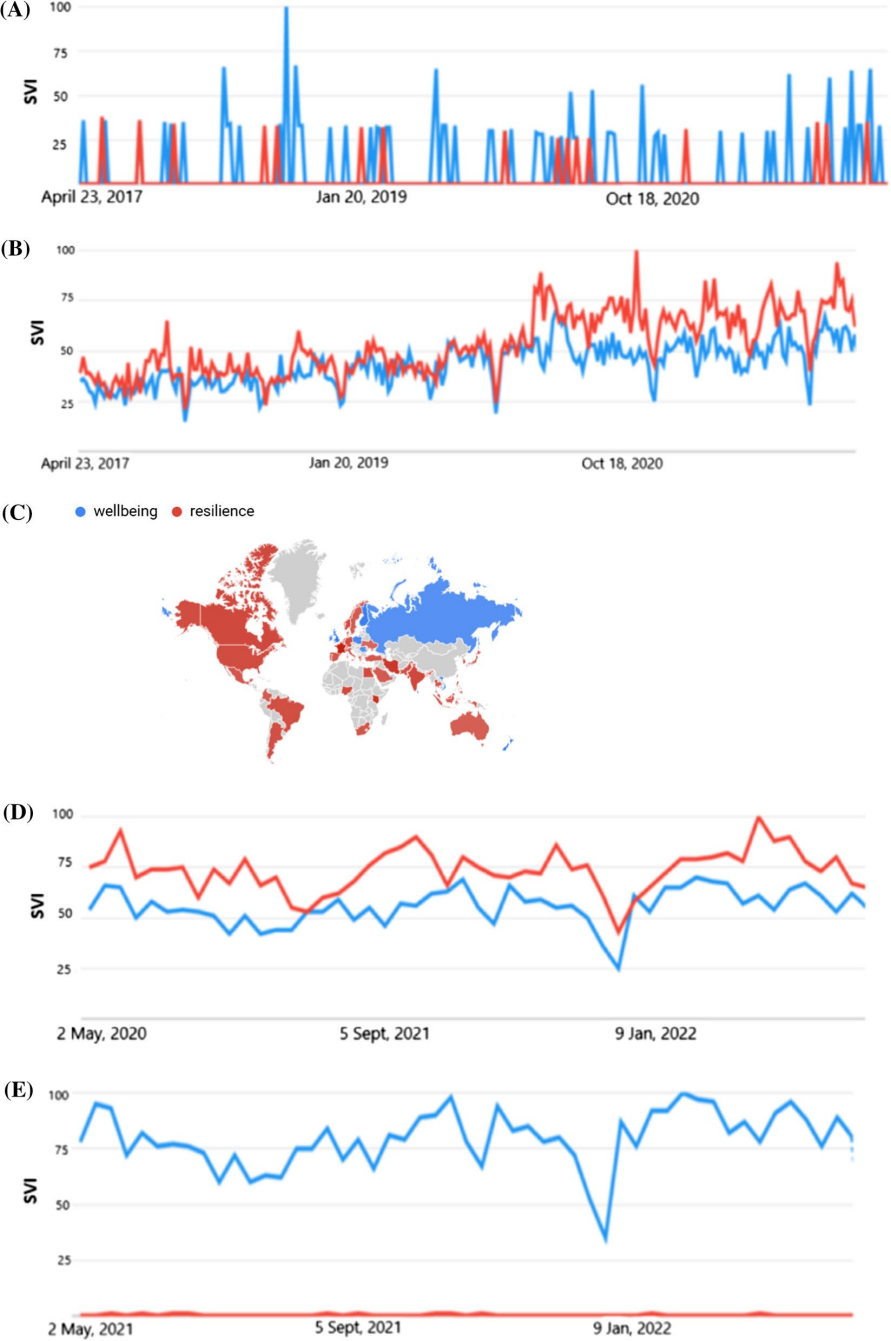
** A** Google trend business and industrial web searches for “worker wellbeing” compared to “worker resilience” for the last 5 years. The y axis is the SVI on an arbitrary scale set by Google from 0 to 100 and the x axis is the time scale. Blue line is wellbeing and red line is resilience. **B** Google trend business and industrial category web searches for “wellbeing” compared to “resilience” for the last 5 years. The y axis is the SVI on an arbitrary scale set by Google from 0 to 100 and the red line is resilience. **C** Global map showing the most popular search interest in “wellbeing” or “resilience”, grey colour suggests low interest or English translation would be required or these terms are not specific for these countries using these search terms. **D** Google trend business and industrial category web searches for “wellbeing” compared to “resilience” for the last 12 months. The y axis is the SVI on an arbitrary scale set by Google from 0 to 100 and the x axis is the time scale. Blue line is wellbeing and red line is resilience. **E)** Google trend business and industrial category web searches for “wellbeing” compared to “psychological resilience” for the last 12 months. The y axis is the SVI on an arbitrary scale set by Google from 0 to 100 and the x axis is the time scale. Blue line is wellbeing and red line is resilience

We next explored GOOGLE web trends for the business and industrial category. Similar trends in searches for both “worker wellbeing” and “worker resilience” was observed across the 5-year time period (Fig. 3a). We noted a drop in SVI over both the pre-covid and COVID period compared to the general category searches.

When the words were truncated to “wellbeing” and compared to “resilience” SVI increased for resilience compared to wellbeing during COVID-19 (see Fig. 3b). Interestingly 40/55 countries had a higher search interest in resilience compared to wellbeing (see Fig. 3c).

We next compared the terms “wellbeing” and “resilience” (Fig. 5a) for the last 12 months to reflect COVID conditions. Interestingly there was a clear increase in SVI for “resilience” compared to “wellbeing” in Fig. 3d. To understand whether there was also interest in the term “psychological resilience” during covid for the business and industrial category this was compared to the term wellbeing (Fig. 3e). We observed a significant reduction in SVI that was sustained close to zero throughout the last 12 months (See Fig. 3e).

## Discussion

In analysing Google Trends data on the key search term wellbeing from the period of April 2017 to April 2022, we found that worldwide online interest in wellbeing had increased during the past two years during COVID. Interestingly not all countries were equal in their interest in wellbeing searches. Additionally, we observed similar trends when we narrowed the search term to “worker wellbeing”. We speculate that increased concerns for worker wellbeing may be associated with countries where there is a social welfare system such as Australia and the UK or there is already a high burden of worker stress due to a history of COVID lockdowns and social distancing [9].

A significant finding in our study was that the search term resilience had a higher SVI when compared to the search term wellbeing for the Google trends category business and industrial. Resilience could cover many aspects of a business as we have alluded to in the induction, including maintaining services or having a more resilient workforce. It was clear however that psychological resilience was not a term of interest in the business and industrial sector during the period of the last 12 months of COVID. Possibly psychological resilience is not a common search phase in the business and industrial category. Alternatively, searches for resilience were believed to be general enough to find relevant information or product interventions that could safeguard the health and wellbeing of business employees during COVID.

We also suggest if there is general interest in resilience as opposed to wellbeing, that resilient training will likely be ineffective if changes in working conditions plus additional factors are not considered to aid worker wellbeing [10]. To our knowledge, this is the first study analysing Google search trends worldwide over time for wellbeing and resilience and relating it also to the Google trends business and industrial category. We have clearly identified a gap where there is more interest in resilience than wellbeing in the business and industrial category. However significantly less interest in “psychological resilience”.

## Conclusion

Google Trends data analysis has shown that there was a reduced tendency to search for key terms, such as wellbeing during covid when compared to resilience for the category business and industrial. COVID has seen a shift in work practices and social economic structural reforms where commercial groups would be vulnerable to both economic and employee stress. There were two interesting observations arising from this study; firstly, psychological resilience as a search term decreased during covid and secondly, there was not equal interest in both wellbeing and resilience searches. Further research is required to ascertain whether there is balanced intertest in resilience and employee wellbeing in business and industrial segments.

### Limitations

The study may have some limitations, we acknowledge that Google is not the only web search engine and terms, and search traffic may vary across different countries and regions. On the other hand, there was an interesting spread of wellbeing and resilience interest across broad areas of the globe. Because of the use of anonymous summary data, we cannot verify whether searches related to products or whether business were specifically searching information for psychological resilience and or interventions for workers or the workplace in general. Finally, there was restricted search to English words (without translation) to ensure we accurately captured the engagement with those specific terms and related definitions. Thus non-English countries will have low search volumes for this reason. However, we have covered a significant area of the Globe to give us trends on search volume to address our original aims. Given these limitations, caution must be given in over interpreting these results and suggest the results rather identify a potential gap for further investigation.

## Data Availability

All available data are present in the manuscript—no additional data is available.

## Abbreviation

SVI: Search volume index
